# Toxicity related to standard TB therapy for pulmonary tuberculosis and treatment outcomes in the REMoxTB study according to HIV status

**DOI:** 10.1186/s12890-019-0907-6

**Published:** 2019-08-14

**Authors:** Conor D. Tweed, Angela M. Crook, Rodney Dawson, Andreas H. Diacon, Timothy D. McHugh, Carl M. Mendel, Sarah K. Meredith, Lerato Mohapi, Michael E. Murphy, Andrew J. Nunn, Patrick P. J. Phillips, Kasha P. Singh, Melvin Spigelman, Stephen H. Gillespie

**Affiliations:** 1 MRC Clinical Trials Unit at University College London, London, UK; 2 University of Cape Town Lung Institute, Cape Town, South Africa; 3 TASK Applied Science, Cape Town, South Africa; 4 Division of Infection and Immunity, University College London, London, UK; 5 TB Alliance, New York, USA; 6 Perinatal HIV Research Unit, Johannesburg, South Africa; 7 Division of Pulmonology, University of San Francisco, San Francisco, USA; 8 The Doherty Institute for Infection and Immunity, University of Melbourne and Royal Melbourne Hospital, Parkville, Australia; 9 University of St Andrews Medical School, St Andrews, UK

**Keywords:** Tuberculosis, Clinical trials, HIV, Adverse events

## Abstract

**Background:**

The phase III REMoxTB study prospectively enrolled HIV-positive (with CD4+ count > 250 cells, not on anti-retroviral therapy) and HIV-negative patients. We investigated the incidence of adverse events and cure rates according to HIV status for patients receiving standard TB therapy in the trial.

**Methods:**

Forty-two HIV-positive cases were matched to 220 HIV-negative controls by age, gender, ethnicity, and trial site using coarsened exact matching. Grade 3 and 4 adverse events (AEs) were summarised by MedDRA System Organ Class. Kaplan-Meier curves for time to first grade 3 or 4 AE were constructed according to HIV status with hazard ratios calculated. Patients were considered cured if they were culture negative 18 months after commencing therapy with ≥2 consecutive negative culture results.

**Results:**

Twenty of 42 (47.6%) HIV-positive and 34 of 220 (15.5%) HIV-negative patients experienced ≥1 grade 3 or 4 AE, respectively. The majority of these were hepatobiliary disorders that accounted for 12 of 40 (30.0%) events occurring in 6 of 42 (14.3%) HIV-positive patients and for 15 of 60 (25.0%) events occurring in 9 of 220 (4.1%) HIV-negative patients. The median time to first grade 3 or 4 AE was 54 days (IQR 15.5–59.0) for HIV-positive and 29.5 days (IQR 9.0–119.0) for HIV-negative patients, respectively. The hazard ratio for experiencing a grade 3 or 4 AE among HIV-positive patients was 3.25 (95% CI 1.87–5.66, *p* < 0.01). Cure rates were similar, with 38 of 42 (90.5%) HIV-positive and 195 of 220 (88.6%) HIV-negative patients (*p* = 0.73) cured at 18 months.

**Conclusions:**

HIV-positive patients receiving standard TB therapy in the REMoxTB study were at greater risk of adverse events during treatment but cure rates were similar when compared to a matched sample of HIV-negative patients.

## Introduction

Infection with the human immunodeficiency virus (HIV) continues to act as a major driver of the global tuberculosis (TB) epidemic [1, 2]. HIV infection results in greater chance of developing active TB by causing a widespread impairment of the body’s immune system [3]. HIV-positive patients have been shown to be more likely to be sputum smear negative [4, 5], and have fewer cavities on chest X-ray [6, 7]. This is thought to reflect the frequently lower bacillary burden in co-infected individuals [8], translating into a delay in the diagnosis of TB in HIV-positive patients [9, 10], with longer time between onset of disease and initiation of treatment leading to worse treatment outcomes [11, 12].

There is an increased risk of toxicity in HIV-positive patients being treated for active TB [13–16], and higher rates of treatment failure and relapse have been described in anti-retroviral therapy (ART)-naïve HIV-positive patients being treated for active TB [17–19]. The overall risk of treatment failure is reduced in the context of adherence to ART [17, 20] and the World Health Organisation has recommended ART as early as possible for all HIV-infected patients since 2016 [21], but despite significant gains in the roll-out of ART globally there are still many areas where treatment coverage is below 50% [22]. Clinicians in settings with limited availability of ART need an accurate understanding of the expected course for patients on treatment, including the incidence of toxicity, to ensure that they react in an appropriate manner to indicators of poor outcome.

We have used the efficacy and safety data collected for patients receiving standard TB therapy as part of the REMoxTB study to obtain a detailed picture of the incidence of treatment-related toxicity and cure rates according to HIV status. The baseline characteristics for HIV-positive patients were compared to HIV-negative patients randomised in the trial, response to treatment was investigated using measured variables, and the nature of toxicity on treatment and the impact on treatment outcomes compared for patients with and without HIV infection.

## Methods

### Baseline characteristics and matched patient sample

The baseline characteristics for all randomised patients across the treatment arms in REMoxTB were compared to determine any significant differences. Characteristics from before the initiation of treatment were collected for HIV-positive and HIV-negative patients. Patients taking ART at screening were screened out, however, files in the dataset relating to concomitant medications were used to determine the number of patients started on ART during the trial and the timing of initiation. ART initiation was at the discretion of the treating physician, in keeping with best practice at the time, and current ART use was an exclusion criteria for patients being screened for the trial.

Coarsened exact matching (CEM) [23, 24] was used to create a population of HIV positive cases matched to HIV negative controls, all treated with standard TB therapy. Standard TB therapy was defined as 2 months of isoniazid, rifampicin, pyrazinamide, and ethambutol followed by 4 months of isoniazid and rifampicin alone. Age, gender, ethnicity and trial site were selected as the matching variables to give the maximum number of matching variables with the greatest number of HIV-positive patients matched.

### Toxicity during treatment

The number of HIV-positive and HIV-negative patients who experienced ≥1 grade 3 or 4 adverse event (AE) was calculated for both the total grade 3 or 4 AEs (as per the DAIDS criteria) and only for those that were considered related to treatment by the reporting clinician. Grade 3 or 4 AEs with and without recorded start dates were included when identifying these patients and events were collated according to System Organ Class (SOC).

### Longitudinal analysis of the matched population

The mean value was calculated for haemoglobin result, patient weight, number of TB symptoms reported and alanine transferase (ALT) result at protocol-scheduled visits for HIV-positive and HIV-negative patients in the matched population. Blood samples were collected at weeks 0, 2, 8, 12, and 17 routinely for all patients. Weight and number of TB symptoms reported was documented for each protocol-scheduled visit during enrolment in the trial. The mean value for each variable was calculated for each scheduled visit and measurements taken at unscheduled visits were discarded as these would tend to occur around adverse events.

### Treatment outcomes

The “first sustained negative culture” was defined as the first negative Mycobacterial Growth Indicator Tube (MGIT) culture with a subsequent negative culture collected a minimum of 1 week later with no positive cultures in between. The median time to sustained negative culture was calculated for HIV-positive and HIV-negative patients in the matched sample, and Kaplan-Meier curves were constructed for the time to first sustained negative culture in both patient groups.

Patients were then assigned into groups based on whether they achieved a microbiological cure. A composite culture status at 18 months, requiring negative cultures on both Lowell-Johnson (LJ) slopes and MGIT culture to be considered culture negative, was available and patients who were culture negative at 18 months were labelled as “cured”. If a patient was lost to follow up, or died before 18 months in the trial, then they were considered cured if they had completed their treatment and had two or more consecutive negative cultures (at different visits) prior to the date that they were last seen. Those patients who were culture positive at 18 months, or who were lost to follow up or died with less than two consecutive negative cultures immediately prior, were considered not cured for this analysis.

### Statistical methods

All data manipulation and analysis was carried out using Stata version 14 (StataCorp, Texas) and statistical significance was set at 5% for all tests. The Chi Square test was used to test for significant differences between sample proportions.

Kaplan-Meier curves were constructed for time to event analyses, and the logrank test was used to test for significant difference in the time to event between groups. Hazard ratios were generated using Cox regression comparing groups where a significant difference was detected using the logrank test and Schoenfeld residuals confirmed the proportional hazard assumption.

Generalised estimation equations (GEEs) were used to investigate the association between HIV status and the longitudinal change in haemoglobin, weight, symptom count, and ALT results using a Gaussian distribution and exchangeable correlation matrix. Weeks after first dose of treatment, a multiple of week number and HIV status (to test interaction) were included as predictors, along with the squared value of the week number.

Zellner’s seemingly unrelated regression [25] was used to build a multivariate model testing the effect of HIV status (as the exposure variable) on the first and last haemoglobin, weight, TB symptom count, and ALT results (as outcome variables). This multivariate regression method uses simultaneous generalised linear regression on a number of outcomes of interest in relation to one or more exposure variables, but there is an assumption that the covariance for the error terms between the individual linear regression models is non-zero. To allow for a more appropriate comparison of variables in the model, the screening value and the result at week 17 were used as all variables had these time points routinely measured.

### Ethics approval and participant consent

The REMoxTB study was carried out with approval from the ethics board at University College London and the local ethics boards at each of the sites where patients were treated. This included approval for the use of data and samples collected in other studies to improve the diagnosis and treatment of tuberculosis. All randomised patients agreed to any data and samples collected as part of the trial being used in further studies to improve the diagnosis and treatment of tuberculosis, as stated on the informed consent form for the study. All the research activities and data collection for the study was compliant with the Helsinki Declaration and the principles of Good Clinical Practice.

## Results

### Baseline characteristics for all randomised patients

The baseline characteristics for all randomised patients in the REMoxTB study are presented in Table 1 according to HIV status. There were 37 of 140 (26.4%) HIV-positive patients and 349 of 1790 (19.5%) HIV-negative patients with no cavitation seen on chest X-ray (*p* value 0.02). However, the proportion of patients with a time to culture positivity in liquid medium (TTP) less than the median value for the total analysis sample was similar irrespective of HIV status: 73 of 140 (52.1%) of HIV-positive and 841 of 1790 (47.0%) HIV-negative patient sputum samples (p value 0.24).

**Table 1:** Baseline characteristics of all randomised patients for REMoxTB according to HIV status. The column percentages included in cells indicate the proportion of total n in for HIV-positive and HIV-negative patients belonging to categories of baseline characteristic. Chi square *p* values comparing the proportions of patients with each characteristic according to HIV status are also provided. *Median time to MGIT culture positive result for all patients = 118 h

n	HIV-positive	HIV-negative	*P* value
	140	1790	–
Gender			
Male	78 (55.7%)	1267 (70.8%)	< 0.001
Female	62 (44.3%)	523 (29.2%)	
Age in Years			
< 25	20 (14.3%)	545 (30.5%)	< 0.001
25–35	60 (42.9%)	546 (30.5%)	
> 35	60 (42.9%)	699 (39.0%)	
Baseline Weight			
< 40	4 (2.9%)	17 (9.6%)	< 0.001
40–45	15 (10.7%)	311 (17.4%)	
> 45–55	50 (35.7%)	716 (40.0%)	
> 55–75	65 (46.4%)	552 (30.8%)	
> 75	6 (4.3%)	40 (2.2%)	
Ethnicity			
Black	128 (92.1%)	734 (41.0%)	< 0.001
Asian	3 (2.2%)	587 (32.8%)	
Caucasian	0 (0.0%)	3 (0.2%)	
Other	8 (5.8%)	8 (5.8%)	
Smoking Status			
Never	70 (50.0%)	800 (44.7%)	0.141
Previous	38 (27.1%)	438 (24.5%)	
Current	32 (22.9%)	552 (30.8%)	
Chest X-ray			
Cavities	80 (57.1%)	1247 (69.7%)	0.015
No cavities	37 (26.4%)	349 (19.5%)	
MGIT TTP*			
≥ Median	67 (47.9%)	949 (53.0%)	0.239
< Median	73 (52.1%)	841 (47.0%)	

### Matched population of HIV-positive and HIV-negative patients assigned to standard TB therapy

Coarsened exact matching (CEM) produced a matched population of 42 HIV-positive cases to 220 HIV-negative controls all receiving standard TB therapy in REMoxTB using the matching variables (see Table 2). There were 4 HIV-positive cases that could not be adequately matched to HIV-negative controls. The L_1_ statistic indicates the measure of global balance between perfect global balance (L_1_ = 0) and complete separation between the matching variables (L_1_ = 1). The multivariate L_1_ statistic was 0.152 which indicated overall low global imbalance. The median CD4+ cell count among the HIV-positive patients was 370 cells per mm^3^ (IQR 307–456). Only 10 HIV-positive patients were started on ART following commencing therapy into the trial at a median time of 180 days, with no patients starting ART in the first 8 weeks of standard TB therapy.

**Table 2:** Baseline characteristics of matched patients in analysis sample from REMoxTB according to HIV status. The column percentages included in cells indicate the proportion of total n in for HIV-positive and HIV-negative patients belonging to categories of baseline characteristic. Chi square p values comparing the proportions of patients with each characteristic according to HIV status are also provided. *Median time to MGIT culture positive result for all patients = 109 h

n	HIV-positive	HIV-negative	*P* value
	42	220	
Gender			
Male	23 (54.8%)	154 (70.0%)	0.053
Female	19 (45.2%)	66 (30.0%)	
Age in Years			
< 25	4 (9.5%)	40 (18.2%)	0.379
25–35	17 (40.5%)	84 (38.2%)	
> 35	21 (50.0%)	96 (43.6%)	
Baseline Weight			
< 40	2 (4.8%)	9 (4.1%)	0.965
40–45	7 (16.7%)	39 (17.7%)	
> 45–55	16 (38.1%)	83 (37.7%)	
> 55–75	15 (35.7%)	83 (37.7%)	
> 75	2 (4.8%)	6 (2.7%)	
Ethnicity			
Black	38 (90.5%)	186 (84.6%)	0.583
Asian	2 (4.8%)	20 (9.1%)	
Other	2 (4.8%)	14 (6.4%)	
Smoking Status			
Never	18 (42.9%)	114 (51.8%)	0.309
Previous	11 (26.2%)	61 (27.7%)	
Current	13 (31.0%)	45 (20.5%)	
Chest X-ray			
Cavities	26 (61.9%)	160 (72.7%)	0.062
No cavities	11 (26.2%)	32 (14.5%)	
MGIT TTP			
≥ Median	22 (52.4%)	118 (53.6%)	0.881
< Median	20 (47.6%)	102 (46.4%)	

### Toxicity during treatment in the matched patient sample

A total of 20 of the 42 (47.6%) HIV-positive patients experienced ≥1 grade 3 or 4 AE, as opposed to 34 of the 220 (15.5%) HIV-negative patients. One or more grade 3 or 4 AE considered to be at least possibly related to treatment was reported by 11 of 42 (26.2%) HIV positive patients and 14 of 220 (6.4%) HIV-negative patients. The majority of these were hepatobiliary disorders that accounted for 12 of 40 (30.0%) events occurring in 6 of 42 (14.3%) HIV-positive patients and for 15 of 60 (25.0%) events occurring in 9 of 220 (4.1%) HIV-negative patients (see Table 3).

**Table 3:** System Organ Classes (SOCs) for the five most frequently reported grade 3 or 4 Adverse Events (AEs) reported by HIV-positive and HIV-negative patients in the matched population during enrolment in the REMoxTB trial. Total number of grade 3 or 4 adverse events of the SOC shown according to HIV status and percentage of total number of grade 3 or 4 AEs in HIV group provided in brackets

Adverse Event SOC	HIV-positive Patients (% of G3+ Events)	HIV-negative Patients (% of G3+ Events)
Hepatobiliary disorders	12 (30.0%)	15 (25.0%)
Respiratory and thoracic disorders	7 (17.5%)	13 (21.7%)
Blood and lymphatic disorders	6 (15.0%)	10 (16.7%)
Infections and infestations	3 (7.5%)	5 (8.3%)
Metabolism and nutrition disorders	3 (7.5%)	4 (6.7%)
Other	9 (17.5%)	13 (16.7%)

**Fig. 1:**
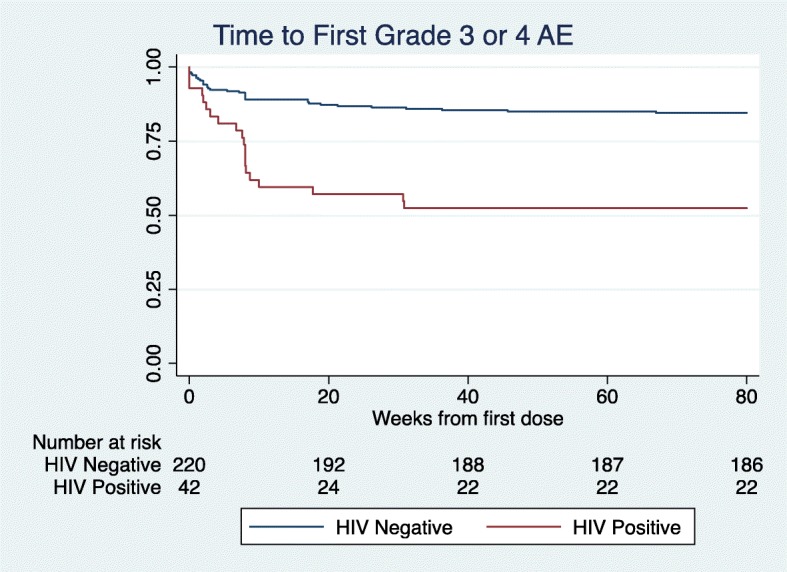
Kaplan-Meier curves demonstrating the time to first related or unrelated grade 3 or 4 adverse event according to patient HIV status in the matched sample. There were 3 HIV-positive and 42 HIV-negative patients who experienced ≥1 grade 3 or 4 adverse event for whom none of the events had an associated start time, and they were excluded from the KM curve. The hazard ratio for HIV-positive patients experiencing ≥1 grade 3 or 4 adverse event was significantly elevated at 3.25 (95% CI 1.87–5.66, p value < 0.01)

The hazard ratio for experiencing a grade 3 or 4 AE for HIV-positive patients was significantly elevated compared to HIV-negative patients at 3.25 (95% CI 1.87–5.66, *p* value < 0.01), and remained significant for related-only grade 3 or 4 AEs (HR 3.77, 95% CI 1.65–8.60, p value < 0.01). Figure 1 presents Kaplan-Meier curves for the time to first grade 3 or 4 AE based on HIV status. The median time to first grade 3 or 4 AE was 54 days (IQR 15.5–59.0) for HIV positive patients and 29.5 (IQR 9–119) for HIV negative patients. This was 56 days (IQR 29–57) for HIV-positive patients and 50 days (IQR 19–56) for HIV-negative patients for related-only events.

### Longitudinal analysis of the matched population assigned to standard TB therapy

The mean haemoglobin at the beginning of treatment was 0.89xLLN and 1.00xLLN at week 17 among HIV-positive patients, and 0.93xLLN and 1.08xLLN at the same time points for HIV-negative patients. The mean weight gain from baseline to week 26 was 3.16 kg for patients infected with HIV and 4.82 kg among HIV-negative patients in the matched sample. HIV-positive patients had a mean weight of 55.81 kg at the beginning of their treatment and 62.05 kg at week 78, compared to a mean weight of 53.61 kg at baseline and 61.19 kg at week 78 in HIV-negative patients.

Table 4 demonstrates the frequency of the 10 most commonly-reported symptoms according to HIV status. There were 21 of 42 (50.0%) HIV-positive patients reporting ≥1 TB-related symptoms at baseline with a mean of 5.19 symptoms reported per patient. Among the HIV-negative patients, 126 of 220 (57.3%) reported ≥1 symptom in keeping with active TB and there was a mean of 4.60 symptoms recorded per patient.

**Table 4:** The ten most frequently reported TB-related symptoms in the matched analysis sample of HIV-positive and HIV-negative patients. The number presented is the number of times a symptom was reported, and patients could not report a symptom more than once at a visit. Symptom count taken from all scheduled visits in the REMoxTB database for these patients, and unscheduled visits have been excluded. Percentages based on the total number of symptoms reported by these patients over the course of the trial. Percentage of all symptoms reported at scheduled visits accounted for by row, according to HIV status, is shown in brackets

TB Symptom	Frequency of Reported Symptoms	
	HIV-Positive (% of symptoms)	HIV-Negative (% of symptoms)
Cough	104 (18%)	555 (19%)
Chest pains	84 (16%)	420 (15%)
Shortness of Breath	76 (13%)	331 (12%)
Fever	73 (13%)	350 (12%)
Night Sweats	76 (13%)	351 (12%)
Weight Loss	63 (11%)	294 (10%)
Haemoptysis	36 (6%)	137 (5%)
Respiratory Abnormalities	13 (2%)	101 (4%)
Loss of Appetite	8 (1%)	82 (3%)
Fatigue	10 (2%)	62 (2%)
Other	21 (4%)	2692 (6%)

The mean ALT result at week 8 was 1.10xULN and 0.68xULN for HIV-positive and HIV-negative patients, respectively. At week 12, the mean ALT was 1.25xULN for HIV-positive patients and 0.61xULN for HIV-negative patients.

Of these four variables, only haemoglobin was significantly associated with HIV status in a generalised estimation equation (coef − 0.05, 95% CI [− 0.098] – [− 0.003], *p* value 0.04). Multivariate regression of the above variables at week 0 and week 17 against HIV status as an exposure variable did not demonstrate any statistically significant associations (see Table 5).

**Table 5:** Regression coefficients from a multivariate model using Zellner’s seemingly unrelated regression to test the effect of HIV infection on the dependent variables measured at Week 0 and Week 17. P values and 95% confidence intervals are provided for the model estimations. The regression coefficient relates to the effect of HIV-positive status on the outcome variable in a linear regression equation, with a negative value indicating a negative effect on the value of the outcome

	Regression Coefficient	95% CIs	*P* value
Haemoglobin			
Week 0 Hb	−0.05	−0.10 - 0.00	0.07
Week 17 Hb	−0.05	−0.10 - 0.00	0.06
Weight			
Week 0 Wgt	2.57	−1.24 - 6.38	0.19
Week 17 Wgt	1.71	−1.98 - 5.40	0.36
Symptoms			
Week 0 Symp	0.04	−0.68 - 0.75	0.92
Week 17 Symp	−0.59	−1.37 - 0.18	0.13
ALT Result			
Week 0 ALT	−0.06	−0.22 - 0.10	0.45
Week 17 ALT	0.00	−0.22 - 0.21	0.97

### Treatment outcomes in the matched patient samples

The median time to the first sustained negative MGIT culture was 84 days (IQR 49–123) for HIV-positive patients and 73 days (IQR 49.5–172) in HIV-negative patients assigned to standard TB therapy. No significant difference was detected in the time to first sustained negative culture between HIV-positive and HIV-negative patients (logrank p value 0.60).

There were 4 of 42 (9.5%) of HIV-positive patients who failed to achieve microbiological cure and 25 of 220 (11.4%) HIV-negative patients in the matched sample (p value 0.73)**.** There were 4 of 38 (10.5%) HIV-positive and 8 of 195 (4.1%) HIV-negative were withdrawn from treatment in the trial and referred to the National Treatment Program (NTP) at a median time of 41.5 days (IQR 19–53) and 130 days (IQR 93.5–150.5), respectively, who then had subsequent negative cultures in follow up and were classed as microbiological cures. “Adverse reaction/toxicity” was the reason given for withdrawal in 3 of the 4 (75.0%) HIV-positive patients and 3 of the 8 (37.5%) HIV-negative patients cured in the NTP. One HIV-positive patient was referred to the NTP after completing standard TB therapy in the trial for further treatment because of disease relapse (and not classed as a microbiological cure); however, none of the HIV-negative patients were treated for relapse in this matched sample.

## Discussion

Our findings demonstrate that ART-naïve, HIV-positive, smear-positive tuberculosis patients with CD4+ counts > 250 cells/mm^3^ display fewer cavities on chest X-ray despite similar sputum bacillary load and are more likely to be anaemic before and at completion of treatment than HIV-negative patients. HIV-positive patients were at greater risk of toxicity during antituberculosis treatment but not at greater risk of failing to achieve microbiological cure than a matched sample of HIV-negative patients.

HIV-positive patients more frequently present with pauci-bacillary disease and extra-pulmonary TB [3, 26, 27] and, while this analysis detected a significant trend for active TB without cavitation, it is noteworthy that the HIV-positive patients in both the total and matched samples did not differ significantly from the HIV-negative patients in the time to positive result (TTP) of MGIT sputum culture at baseline. This suggests comparable burden of disease in both groups and argues against the accepted maxim that HIV-positive patients present with less of a bacillary load. While there should be a degree of caution before applying these results to the wider HIV-positive population, as there is a spectrum of disease severity predominantly according to the CD4+ count [28, 29], some conclusions can be drawn. These findings confirm the importance of testing all TB patients for HIV infection [25]. This is most relevant in resource-limited settings, where constrained access to HIV testing can lead to testing only cases with the highest clinical suspicion. However, conclusions should be taken in light of the requirement that patients were smear positive at screening as this will have acted to introduce bias by selecting out both HIV-positive and HIV-negative patients with a lower bacillary burden.

The World Health Organisation (WHO) recommends routine screening of HIV-positive patients for active TB using a clinical algorithm of one or more of the following symptoms: cough, fever, weight loss, or night sweats [30, 31]. In a meta-analysis reviewing the individual patient-level data of HIV-positive patients with pulmonary TB, the best-performing diagnostic strategy was the presence of any one of these four symptoms [32]. This meta-analysis demonstrated a sensitivity of 79% and a specificity of 50% after including data for over 8000 patients, and the negative predictive value was 90% at 20% prevalence of TB among HIV-infected individuals.

In the REMoxTB study chest pains, fever, cough, and breathlessness were four of the most common symptoms reported in the HIV-positive patients. These four symptoms collectively made up only 61% of the symptoms reported, and weight loss accounted for just 14% of the screening symptoms reported among the HIV-positive group. The GeneXpert platform allows for the rapid identification of *M tuberculosis* in sputum (and other bodily fluids) with a sensitivity of up to 75% even in cases of sputum smear-negative pulmonary TB [33], and this also provides molecular results for the rifampicin resistance profile of the organism. The WHO now recommend GeneXpert as a first-line method for diagnosing active TB [34]; however the infra-structure required to support this technology has acted as a barrier to widespread implementation. The symptom data presented here shows a poor sensitivity for the WHO-recommended screening approach among these selected HIV-positive patients (with higher CD4+ counts), and therefore lends support to the argument that infra-structure development to allow the use of existing, validated diagnostic technology in resource poor environments remains a priority [35].

HIV-positive patients in this sample were treated with standard TB therapy lasting 6 months, and there was no significant difference in the proportion of patients who were cured 18 months after commencing therapy. The most recent guidance from the Infectious Diseases Society of America (IDSA) recommends extending TB treatment to 9 months in cases where HIV-positive patients are not taking ART to reduce the risk of treatment failure or relapse, and to treat with standard TB therapy for 6 months if a patient is receiving ART [36]. While the findings presented here are encouraging, with only one case of relapse among the HIV-positive patients meriting re-treatment following standard TB therapy, these results must be interpreted with a degree of caution. The patient sample was small and there was a high cure rate overall on standard TB therapy, and even in the context of a matched population there is a risk that a difference will be missed. Furthermore, previous work has demonstrated a significant association between poor treatment outcomes and adverse events in this study population [37].

Coarsened exact matching (CEM) was adopted to address the limitations of the small numbers in this analysis, however there is still a risk that effect size will be affected due to undetected sampling bias. A further limitation to this analysis is the exclusion of HIV-positive patients with a CD4+ count less than 250 cells/mm^3^, as this means that the rates of adverse events should be considered to represent a favourable scenario for patients not receiving ART. This should also be noted when considering the data on clinical change over time and microbiological outcomes. Unfortunately, this means that broader conclusions about ART-naïve TB-HIV patients cannot be drawn from this analysis. ART has been conclusively shown to improve TB-HIV co-infected patients’ outcomes [38, 39], and this paper does not disagree with the need for early ART in these patients despite the reassuring treatment outcomes.

## Conclusions

The increased risk of adverse events associated with HIV infection has implications for clinical monitoring of these patients and this work which studies a patient cohort with a relatively high CD4 count, confirms the need for clinicians to ensure that TB programmes focus on this high-risk group even in cases where the CD4+ is preserved. The treatment outcomes among the HIV-positive and HIV-negative patients were similar with 6 months of standard TB therapy in this matched population, and those patients who required longer durations of therapy were identified early in treatment. TB therapy is particularly toxic for HIV-positive patients, and this would suggest that those patients performing well clinically do not need to be exposed to a longer duration of drugs and the concomitant risk of further toxicity. In future Phase III TB trials it will be critical that recruitment and monitoring of HIV-patients is undertaken with sufficient accuracy to translate into real clinical benefit for this often under-served patient population.

## Data Availability

The data that support the findings of this study are available from MRC CTU at UCL but restrictions apply to the availability of these data, which were used under license for the current study, and so are not publicly available. Data are however available from the first author (CD Tweed) upon reasonable request and with permission of MRC CTU at UCL.

## Abbreviations

AEs: Adverse events
ALT: Alanine aminotransferase
ART: Anti-retroviral therapy
CEM: Coarsened exact matching
GEE: Generalised estimation eq.
HIV: Human immunodeficiency virus
IDSA: Infectious Diseases Society of America
IQR: Interquartile range
LJ: Lowell-Johnson
MGIT: Mycobacterial growth indicator tube
NTP: National treatment program
SOC: System organ class
TB: Tuberculosis
TTP: Time to culture positivity in liquid medium
WHO: World Health Organisation
